# Professionals’ responses to the introduction of AI innovations in radiology and their implications for future adoption: a qualitative study

**DOI:** 10.1186/s12913-021-06861-y

**Published:** 2021-08-14

**Authors:** Yaru Chen, Charitini Stavropoulou, Radhika Narasinkan, Adrian Baker, Harry Scarbrough

**Affiliations:** 1 Centre for Healthcare Innovation Research, City, University of London, London, UK; 2 School of Health Sciences, City, University of London, Northampton Square, London, EC1V 0HB UK; 3 Bayes Business School, City, University of London, 106 Bunhill Row, London, EC1Y 8TZ UK

**Keywords:** AI innovation, Professional, Radiologist, Radiographer, Perception, Adoption, NHS

## Abstract

**Background:**

Artificial Intelligence (AI) innovations in radiology offer a potential solution to the increasing demand for imaging tests and the ongoing workforce crisis. Crucial to their adoption is the involvement of different professional groups, namely radiologists and radiographers, who work interdependently but whose perceptions and responses towards AI may differ. We aim to explore the knowledge, awareness and attitudes towards AI amongst professional groups in radiology, and to analyse the implications for the future adoption of these technologies into practice.

**Methods:**

We conducted 18 semi-structured interviews with 12 radiologists and 6 radiographers from four breast units in National Health Services (NHS) organisations and one focus group with 8 radiographers from a fifth NHS breast unit, between 2018 and 2020.

**Results:**

We found that radiographers and radiologists vary with respect to their awareness and knowledge around AI. Through their professional networks, conference attendance, and contacts with industry developers, radiologists receive more information and acquire more knowledge of the potential applications of AI. Radiographers instead rely more on localized personal networks for information. Our results also show that although both groups believe AI innovations offer a potential solution to workforce shortages, they differ significantly regarding the impact they believe it will have on their professional roles. Radiologists believe AI has the potential to take on more repetitive tasks and allow them to focus on more interesting and challenging work. They are less concerned that AI technology might constrain their professional role and autonomy. Radiographers showed greater concern about the potential impact that AI technology could have on their roles and skills development. They were less confident of their ability to respond positively to the potential risks and opportunities posed by AI technology.

**Conclusions:**

In summary, our findings suggest that professional responses to AI are linked to existing work roles, but are also mediated by differences in knowledge and attitudes attributable to inter-professional differences in status and identity. These findings question broad-brush assertions about the future deskilling impact of AI which neglect the need for AI innovations in healthcare to be integrated into existing work processes subject to high levels of professional autonomy.

## Introduction

Increasing demands on radiology departments together with rapidly evolving technologies generating vast and complex datasets have positioned radiology as a priority area for the deployment of Artificial Intelligence (AI)-based innovations [1]. Such innovations are increasingly demonstrating their potential capacity to improve triage, diagnosis and workflow within this field. Such support is arguably needed. In NHS England, 44.9 million imaging tests were performed in 2019 [2], and there are ever-increasing pressures on radiology departments to perform fast, high throughput assessment of medical images. At the same time, the UK, along with many other countries worldwide, is experiencing an acute shortage of radiologists [3].

The pattern recognition and machine learning capacities of AI technology suggest that radiology represents one of the most promising areas for the application of AI in medical practice [1]. AI’s automatic recognition of complex patterns in imaging data provides ‘quantitative, rather than qualitative, assessments of radiographic characteristics’ [4]. For example, an AI algorithm for diagnosis in mammography in breast cancer has been used successfully in identifying imaging abnormalities in an accurate and timely manner [5], while another AI algorithm in thoracic cancer was shown to equal or even exceed radiologists’ performance for chest X-ray single anomalies [6, 7].

The introduction of such AI-based innovations into the radiology field, however, is accompanied by the widespread and sometimes extravagant claims which are being made about the technology’s wider impact on society [8]. Much of this ‘hype’ around AI is driven by simplistic assumptions based on the potential of the technology alone [7]. In contrast, a number of studies are beginning to highlight the challenges involved in translating AI innovations into healthcare settings. These studies suggest that such challenges are not trivial, and are not reducible to technical concerns over datasets and infrastructure, though these do pose a significant barrier [9, 10]. Rather, they often relate to the human and behavioural aspects of translation, and the need to make complementary changes in work practice to accommodate the use of AI innovations [11]. In fact, it has been argued that the biggest adoption challenge for AI is not a lack of data or analytics but the behaviour of healthcare professionals [12].

These studies reinforce experience with previous forms of innovation [13], in suggesting that healthcare practitioners are likely to play a key role in the adoption of AI technologies in the NHS. Their positive contribution here may include acting as champions for new technologies, promoting adoption within their healthcare organisations, and being receptive to new skills and changes in working practices [14]. Conversely, from a negative standpoint, they may be slow or reluctant to see the benefits of AI, unwilling to change their skills and practices, and may demonstrate resistance to adoption [15]. Internationally, the empirical evidence to date has shown a mixed response on the part of healthcare professionals to AI innovations. Although they are seen to be largely positive about using AI tools [16, 17], they are also concerned about job insecurity resulting from AI implementation [17].

In the field of radiology itself, the work is currently divided between two distinct professional groups. Medical imaging for the detection of diseases is performed by trained radiographers, and is then visually assessed by qualified radiologists. These groups are regulated by different bodies and perform different roles. Radiographers have the task of physically conducting imaging scans on patients, while radiologists are responsible for interpreting these scans to diagnose conditions and decide on treatments. The two groups normally work interdependently, since they have different roles and responsibilities and are differentiated by status and expertise.

Our framework for identifying the implications of these professionals’ responses to the introduction of AI into radiology is provided by the ‘innovation-decision process’ framework [18]. This is based on Rogers’ theory of innovation diffusion which defines innovation as ‘an idea, practice, or object that is perceived as new by an individual or other unit of adoption’ [18]. Rogers’ approach is helpful in questioning the technological determinism which is often implicit in the ‘hype’ generated by technological advances. By framing the introduction of new technologies, such as AI, in terms of their novelty, we are sensitized to the uncertainty which they pose for those deciding on their adoption. The ‘innovation-decision process’ framework is an analytical tool for addressing the way individuals and organizations respond to that uncertainty, providing valuable insights into what these responses may mean for receptiveness towards the future adoption of innovations. It does so by relating such responses to the important pre-adoption states of ‘knowledge’ and ‘persuasion’. *Knowledge* of the innovation arises when individuals learn of its existence, and gain some understanding of how it functions. *Persuasion* occurs when individuals form a favourable or unfavourable attitude towards the innovation.

These pre-adoption states are influenced in part by the way information is communicated to individuals, for example by intermediary groups, innovators and the media. They also reflect the information seeking and processing activities of individuals as they seek to reduce uncertainty about an innovation. They do not unfold in a linear fashion but evolve cumulatively over time as more information is acquired. For example, individuals typically rely heavily on their social networks to gain knowledge about a particular innovation [18]. Their search is also influenced by their existing roles, needs and interests [19]. Likewise, the formation of attitudinal responses to an innovation is not a ‘tabula rasa’. It may be highly influenced by previous experience, especially knowledge of what are perceived to be similar innovations [20].

The aim of our study is to explore the knowledge, awareness and attitudinal responses related to AI amongst professional groups in radiology, and to analyse the implications for the future adoption of these technologies into practice. In this regard, our study could help to provide insights on the relative receptiveness or resistance which key professions in the NHS may display towards the future deployment of AI innovations in radiology.

## Methods

### Study design

To investigate radiology professionals’ responses to AI we carried out a qualitative empirical study encompassing 26 health professionals from the breast screening units of five NHS organisations in England between 2018 and 2020. Our sampling focussed on these units since they are expected to be amongst the early adopters of AI technology in the NHS due to the pre-existing support provided by a national programme encompassing shared datasets, infrastructure and governance. The introduction of AI innovations was thus viewed as a more imminent prospect in this setting. At the same time, a sample of breast screening units ensured greater comparability in terms of shared practices and work roles, supporting the development of more generalizable findings. To support this aim, we also sought variation in size and geographical locations across the five units selected.

We utilised both semi-structured interviews and focus group methods to explore the professionals’ responses to the introduction of AI innovations in radiology. Following other researchers, the combination of methods serves to triangulate the observations and discussions from focus groups with interviewees’ accounts [21].

Semi-structured interviews were conducted either face-to-face or via telephone. Participants were recruited using a snowball sampling technique, initiated with contacts from conferences/events related to AI in radiology. Our sample of interviewees included 12 radiologists and 6 radiographers. Interviews lasted between 40 min and 2 h and were transcribed verbatim. The interview guide was designed by three of the authors prior to the study and was informed by a thorough review of the literature. We explored with interviewees their understanding of the AI technology that might be introduced into their work setting, including their perceptions of the associated risks and benefits for patients and practitioners, and the likely challenges posed for adoption in their department. Detailed interview questions included: (1) interviewee’s job, organisational role, and professional experience; (2) activities involved regarding the introduction of AI or/and its potential adoption; (3) perceived risks and benefits for patients/practitioners; (4) how AI innovation differs from previous technological innovations in the field; (5) likely challenges for AI innovation adoption in the department/unit; (6) how differently would radiologist and radiographer view/adopt AI innovation and why; (7) information sources that influenced one’s views about AI in their field.

In addition to the interviews, we addressed these topics through a focus group convened with 8 radiographers drawn from the fifth participating NHS organisation. The manager of the breast unit helped organise this focus group with all radiographers at work that day. The focus group was conducted in early 2020 and carried out face to face with two of the authors present. Although the focus group was not recorded, one of the authors led the discussion while the other took detailed notes of the meeting, which was subsequently used as part of the dataset. The focus group participants were not aware of the findings of the individual interviews.

### Data analysis

Three of the authors were engaged in data analysis, which combined elements of inductive and deductive coding and theme development [22]. In line with this analytic approach, our scrutiny of the data was an iterative and reflexive process, with our reading and re-reading of the data being guided both inductively, and by the Rogers ‘innovation-decision process’ framework. When there were divergences in the codes and themes developed, the three authors went back to the data and discussed these further until differences in interpretation were resolved and consensus was reached [21]. Guided by Roger’s framework [19], we focused on the two pre-adoption states: ‘knowledge of’ and ‘attitudes towards’ AI innovation. The interviews were analysed separately for each group, and the data were coded to identify common themes within each state in a way that would allow us to identify commonalities and/or differences between the two healthcare professional groups, eventually reaching data saturation.

## Findings

Relating our findings to the innovation-decision process framework, we can observe a number of expected factors influencing the way these professionals acquired knowledge of, and formed attitudes towards the prospective introduction of AI innovations. In particular, we note the importance of professional and social networks in communicating information about such innovations, and the importance of existing work roles in anchoring the professionals’ attitudinal responses. As outlined in Table 1, and further detailed below in terms of knowledge and attitudes, these factors help to explain some commonalities in the two groups’ responses to the advent of AI in their field. This was especially salient in relation to the importance which both groups ascribe to workforce shortages as a driver for adoption, reflecting their shared experience of the work pressures in radiology departments. At the same time, these factors are also associated with differences in the groups’ knowledge and attitudes, due to their differential access to information. These differences are further accentuated by the mediating impact of their distinctive professional identities and relative status positions.

**Table 1:** Professional responses to the introduction of AI in radiology

	Knowledge	Attitudes	Overall responses
**Radiologists**	Greater bandwidth for information on AI due to professional networks and industry interactions. More nuanced awareness of the potential applications of AI.	Role related benefits perceived around discarding menial tasks Acceptance of implications for risk and responsibility. AI not viewed as a threat to professional control and autonomy.	Greater confidence in their ability to shape the future use of AI innovations. AI viewed more as an opportunity – a new partner in practice.
***Common areas of response***	*Awareness of the potential role of AI innovations in mitigating skill shortages.* *Relate AI to previous generations of technology, especially CAD (Computer Aided Detection).*	*Progressive attitudes towards the use of new technologies in practice.* *Professional identities linked to adapting to new forms of technology.*	*Common responses around accepting the introduction of AI innovation to alleviate currently experienced workforce shortages.*
**Radiographers**	Information from a narrower range of sources; more constrained by everyday job demands and more reliant on localized personal networks	AI viewed as potentially deskilling of their roles, and possibly undermining job security. Greater concern over the implications for risk and responsibility.	Greater uncertainty about the implications of AI for their work roles. Concern about the potential impact on their skills.

Overall, as explained on Table 1, although both groups saw their professional identities as involving a progressive attitude towards new technologies, radiologists were much more confident of their ability to shape the introduction of AI to the benefit of their skills and professional development, while radiographers expressed greater concern about the potential impact of AI on their skills.

### Knowledge of AI innovations

We found that radiologists had greater knowledge of the potential applications of AI than radiographers. This was derived from their professional networks, conference attendance, and also contacts with industry developers of AI. One radiologist comment showed a range of possible interactions they were exposed to: *‘The Royal College is making an effort. BIR (British institute of Radiology) is making an effort. These are the two national bodies, abroad the RSNA, and the AI exhibit there is superb, the sheer amount of companies there, and we get to see them all’ (Unit 1, Radiologist 4).*

Their attitudes were also informed by their work roles, including experience of previous generations of technology such as CAD (Computer Aided Detection): *‘Everyone who is interested in AI will compare it to CAD, and think that AI is another form of CAD. CAD worked, but CAD was not a self-evolving, self-learning algorithm. You fed CAD data and it performed within that scope’ (Unit 1, Radiologist 4).*

As this comment shows, radiologists were inclined to frame AI as a positive improvement compared to existing tools. This may also have reflected a strong identity as a professional group willing to embrace the opportunities afforded by new technologies: *‘Generally my impression of radiologists on the whole is that they are a forward-thinking bunch. We adopt technology very quickly’ (Unit 3, Radiologist 3).*

In contrast, we observed some interesting differences when analysing the radiographers’ interviews. There was a relative lack of knowledge of AI on their part, with more limited access to the professional and industry networks accessed by radiologists. One commented, for example:

*‘In journals we get which are radiography based, I haven’t seen anything…I don’t have access to the radiologist’s websites and things. You don’t qualify for their (radiologist) status to get into theirs, unless you pay their fees and get into their websites’ (Unit 1, Radiographer 1).* This was also confirmed by radiographers from the focus group, where one of the authors noted in the field note, *“although there was general interest in AI innovation in their work, radiographers from unit 5 claimed that they lack exposure to how AI actually works in their daily practice and are constrained in access to information.” (Unit 5, focus group).*

Although radiographers were not unaware of AI and its potential implications to their work, there was great reliance on local networks for information: *‘I am aware that there’s quite a lot going on, because obviously I’ve got colleagues already involved in AI, someone like Ross (anonymised name)’ (Unit 3, Radiographer 4).*

Radiographers also mentioned that they were too busy with their day-to-day jobs to have the capacity to think about AI innovation or find time to read about relevant AI innovation or be personally involved in research projects related to AI innovation. One commented, for example: *‘I think people are so busy doing their jobs that they don’t have the time’ (Unit 2, Radiographer 3).*

### Attitudes towards AI innovations

In relation to the prospect of AI being introduced into their work, we found some common ground in the responses presented by our two professional groups. Common concerns arose primarily from their work roles where ongoing workforce shortages in both professional domains were identified as a significant issue in relation to the adoption of AI. These shortages were seen as a strong argument in favour of the deployment of AI. A typical comment from a radiographer was as follows: *‘AI will free up staff to do other things, in clinics or organise their workloads better in terms of audits. We conduct a lot of audits here as well. I think it will be mostly for that.’ (Unit 2, Radiographer 2).* Similarly, a radiologist commented: *‘We’ve got a workforce crisis so I’ll tell you that the reason that AI is gaining support from Public Health England and gaining momentum is because of the workforce crisis’. (Unit 4, Radiologist 7)*

Both radiologists and radiographers identified changes that AI technology might bring to their professions and argued that their roles had continually been evolving with technological change. As one of our radiographers argued: *‘Job roles and professions have been changing in healthcare over the past 20–40 years…That is what technology brings…Basically, it is all mutating’ (Unit 2, Radiographer 2).* In the same vein, a radiologist commented: *‘In the last 10, 20 years we’ve evolved a huge deal from looking at films in a box to using computers…Our roles will be different with AI coming in’ (Unit 3, Radiologist 3)*.

However, radiologists and radiographers displayed different attitudes when talking about the potential impact of AI technology on their skills, training and development opportunities, and their professional accountability. Radiologists expressed few concerns about the potential for AI to automate their work or affect their skill development, believing that the high-level and varied character of roles made them less vulnerable to such deskilling. Rather, they saw AI innovations as potentially helping to eliminate the more routine aspects of their work, allowing them to focus on more interesting and challenging tasks. This is shown in one of the radiologist’s comments: *‘There is an opportunity for radiologists who have domain knowledge to get involved, lend their expertise to the development of automated tools, relieve some of the menial tasks, more repetitive tasks, the simpler tasks’ (Unit 3, Radiologist 3).*

Radiologists were also less concerned about the implications of AI innovations for their professional accountability in relation to diagnosis and clinical decision-making. They felt confident that their host unit would be able to put in place the relevant policies and mechanisms to safeguard individual professionals if there were challenges to diagnostic methods incorporating AI.

In contrast, radiographers were generally concerned about the potential impact that such AI innovations might have on their skill development, as well as the challenges they might bring to their practice and decision-making. As one commented: *‘I am a little dubious about computerised things. I think people can become too reliant on them and then skills can start faltering.’ (Unit 1, Radiographer 1)*.

Radiographers were also more concerned about the possibility of diagnostic errors due to AI technology, and had less confidence in its overall capability. For example, one of the radiographers observed: *‘I still have my doubts about how sensitive technology can be and you really have to prove it to me before I actually accept it… You need the data to prove that there is a worthwhile system to start.’ (Unit 1, Radiographer 1)*.

What also emerged from our interviews was the way members of each professional group differentiated themselves by reference to the other. Thus, the radiologists in our sample argued that AI technology might be more challenging for radiographers than radiologists, because radiologists are more accustomed to handling risk as a profession and could therefore embrace AI technology more positively. They also presented their roles as encompassing a wider range of tasks than those of radiographers, making their work less vulnerable to potential capture by AI technology. They suggested that as a group radiographers would be more challenged by the introduction of AI because they were more comfortable conforming to procedures and were less willing to take risks. Conversely, radiographers presented themselves as being lateral thinkers and more open-minded, which allowed them to be receptive to new AI technology. Interestingly, they inverted the radiologists’ claims about receptiveness to AI. The radiographers’ view was that that their willingness to follow procedures would make them more accepting of the inclusion of AI technology in the work process. They saw radiologists as a group being more protective of their roles, and more resistant to the possible challenge which AI’s diagnostic functions might present to their decision making autonomy.

## Discussion

Our findings highlight the pre-adoption state of knowledge and attitudes relating to AI amongst these two professional groups. Our qualitative research approach helps to explore nuances in these groups’ responses which relate to their professional experience, roles and identity, and which go beyond the binary distinction between positive and negative attitudes towards AI highlighted in other work [17]. Specifically, we noted how both groups’ responses were shaped by the experience of workforce shortages in the radiology field in the UK. This common experience underpinned a more receptive stance towards the prospective adoption of AI. Similarly, both groups drew on past experience to identify the benefits of new technologies and the ability of their professions to adapt to change.

However, receptiveness towards AI can vary from acceptance of the perceived inevitability of change to positive enthusiasm for such change. In this respect, we found that there were differences in attitudes towards AI between the two groups, with radiologists tending to view AI technology as affording an opportunity for professional development, whereas radiographers were more reticent in orientation, highlighting the possible threat which AI posed to their roles. Although it is not possible to disentangle the causes of such differences in attitudes, in our study we found that they were associated with differing levels of knowledge and awareness around AI exhibited by these groups, as well as distinctive features of their respective roles, professional status and identities.

In relation to differences in knowledge and awareness, we found that radiologists’ professional networks afforded them a more nuanced understanding of the possible scope of AI innovations in practice, and that this helped to limit their concerns around future de-skilling. Instead, there was a recognition of the possible benefits of automating more routine tasks, and possible up-skilling of their roles overall. In contrast, radiographers had acquired less detailed knowledge about possible AI applications, and their networks were less extensive. They had greater role concerns around possible de-skilling and job insecurity, and were also sensitive to the risks posed by the use of AI in diagnosis.

The more cautious stance which radiographers exhibited towards AI seems to be linked not only to their more limited knowledge of AI and immediate role concerns, but also to their professional identity and status relative to radiologists. As we noted, these groups, though interdependent in their work, also maintain carefully differentiated professional identities. While both groups saw their profession as progressive and as embracing new technology, the radiologists in our sample seemed more confident of their ability to champion or control the adoption of AI-based innovations in their practice. This seems to reflect the relative work autonomy they currently enjoy, and their higher professional status and identity as qualified clinicians who have benefitted from an extensive medical education, socialisation with peers, and career development [23, 24]. In contrast, radiographers fall into the category of ‘paraprofessionals’ [25–27] who are delegated to carry out a particular set of tasks and to assist professional groups. As such, this group is subject to tighter performance measures and subject to more intensive managerial control [28].

Although studies of professions suggest that both these groups have an incentive to view the introduction of AI as an opportunity to expand their skills and control of work processes [29], the radiologists in our sample seemed better equipped to adopt this more proactive stance towards AI given their greater knowledge and more extensive networks, as well as their relative professional status and autonomy. Certainly, they viewed AI as less of a threat, and were less concerned about its implications for their present roles and responsibilities.

In summary, our findings suggest that professional responses to AI are linked to existing work roles, including concerns around skills development and workforce shortages, but are also mediated by differences in knowledge and attitudes attributable to inter-professional differences in status and identity. These findings provide valuable insights into the possible future deployment of AI in radiology and healthcare more generally. Specifically, they question broad-brush assertions about the future deskilling impact of AI on professional groups [30]. Such views tend to focus on the potential of the technology, and neglect the way in which AI innovations need to be integrated into existing work processes which are subject to high levels of professional autonomy and control. This is especially the case in healthcare, where inter-professional collaboration is critical to the patient experience and quality of service.

Equally, our findings suggest that the adoption of AI in practice is not a question of either blanket acceptance or resistance on the part of professionals, but will be dependent on the extent to which these professional groups engage positively and knowledgeably with the use of AI innovations within their own settings. Where such use is dependent on different groups working together, as in radiology, our study suggests that inter-professional differences in knowledge and attitudes may create tensions between more opportunity-driven and more defensive responses to the reality of AI use. As our findings show, the status and identity differences which help underpin these responses are persistent, and may therefore play a role in the determination of future work processes based on AI.

By highlighting the pre-adoption responses of professionals in the radiology field, these findings have important implications for the wider debate on the role played by professional groups in the introduction of AI into healthcare. As noted previously, that role may be pivotal in shaping the way AI innovations are integrated into existing processes and work practices [11, 12]. Professionals have the capacity to act both positively and negatively towards the advent of AI, either championing [14] or resisting [15] its introduction. By focussing on pre-adoption responses, our study helps to shed some light on the motivating factors in play behind such stances. As we show, professionals in the radiology field are not ‘Luddites’ who will unthinkingly obstruct the progressive potential offered by AI. These groups are themselves a product of technological change, and in common with other healthcare groups [16, 17], affirm a progressive ‘forward thinking’ stance towards it. At the same time, our study shows that they are capable of more sceptical responses to the over-inflated claims made for AI’s potential [7, 8, 12], recognizing that its benefits, for example in addressing workforce shortages, may be tempered by other less desirable effects, including changes in their own skill-sets.

While our research confirms the mixed response to AI seen in previous studies of healthcare workers generally [16, 17], these findings provide a deeper understanding by showing how the sensitivities of such workers towards AI’s deployment vary according to differences in their work roles and professional identities, such that AI may be seen as an opportunity for professional development by one group, and as a potential threat by another. Our study also makes clear, however, that neither these responses, nor the inter-professional differences that underpin them, are fixed and unchanging. The relative differences that we found in the knowledge and awareness of AI innovations between radiologists and radiographers actually underline the scope for communication and education to influence the pre-adoption state of professional groups. Increasing knowledge and awareness in this way can help to orient such groups more effectively towards the real world implications of AI innovation, countering the more damaging effects of the ‘hype’ around this technology [7, 8, 12].

These findings on professionals’ responses gain added significance from the distinctive features of AI compared to other technologies. AI is emerging as a potential paradigm shift in the organization of work and a particular challenge to the decision-making autonomy of diverse professional groups whose expertise is, for the first time, vulnerable to forms of automation and de-skilling previously confined to lower-skilled workers [30]. With the introduction of AI innovations, certain core elements of professionals’ work risk being replaced by increasingly capable AI innovations. Some commentators are already speculating that this will lead to the dismantling of the traditional professions [30]. On the other hand, the prospect of the delegation of decision-making authority from humans to machines [31], or the outright substitution of fallible or scarce human expertise by more reliable AI tools [32], leaves others more sceptical. Some argue, for instance, that the context-sensitive intelligence and tacit knowledge of professionals still trumps the narrowly data-driven capabilities of AI systems [33].

These arguments resonate strongly with the pre-adoption attitudes highlighted by our findings, with radiologists in particular showing confidence in the indispensability of their professional expertise and therefore their ability to shape the future use of AI.

### Strengths and limitations

The strength of our study is utilising qualitative research methods, especially in-depth semi-structured interviews, to explore professionals’ responses to the introduction of AI innovations in radiology and their implications for future adoption. The qualitative case study method is most suited to providing a detailed understanding of the issues concerned, and explicating the differences among distinctive professional groups. We are also able to explore the pre-adoption stage of AI innovation, which is less well studied in the innovation literature. However, our study is not without limitations. In particular, it is constrained by the number and distribution of interviews in our sample. It would have benefitted further from a larger number of interviews, and greater representation from radiographers. However, this limitation does not affect the validity of the results, with our detailed analysis serving to produce trustworthy outcomes. This limitation is also compensated to some degree by the inclusion of a focus group of radiographers. This helped to balance our data gathering across professional groups, and also increased the data richness of our qualitative approach. Also, as noted, focussing our sample on breast screening units supported that approach by ensuring greater comparability in terms of shared practices and work roles.

## Conclusions

Radiology is at the cutting edge of the introduction of AI innovations into healthcare. Radiology professionals therefore have a major role to play in the adoption of AI, but as yet we have limited knowledge of their responses to this emerging technology, or their implications for its future use. To address this lack, our study focussed on the pre-adoption states of radiology professionals in NHS England, specifically their knowledge and attitudes in relation to AI. Importantly, the delivery of radiology services is dependent on the collaboration between two professional groups marked by differences in status and identity. We found that these groups shared some common understanding on the value of AI as a solution to workforce shortages, but that they also exhibited significant differences in their attitudes, between viewing AI as an opportunity, versus taking a more defensive or sceptical stance. Our analysis highlights the implications of these responses for the future deployment of AI in radiology, particularly the potential tensions which they imply for the development of new AI-based working arrangements between the groups.

## Data Availability

The datasets used and/or analysed during the current study are available from the corresponding author upon reasonable request.
